# Rapid Microbial Sample Preparation from Blood Using a Novel Concentration Device

**DOI:** 10.1371/journal.pone.0116837

**Published:** 2015-02-12

**Authors:** Anna K. Boardman, Jennifer Campbell, Holger Wirz, Andre Sharon, Alexis F. Sauer-Budge

**Affiliations:** 1 Center for Manufacturing Innovation, Fraunhofer USA, Brookline, Massachusetts, United States of America; 2 Department of Mechanical Engineering, Boston University, Boston, Massachusetts, United States of America; 3 Department of Biomedical Engineering, Boston University, Boston, Massachusetts, United States of America; Naval Research Laboratory, UNITED STATES

## Abstract

Appropriate care for bacteremic patients is dictated by the amount of time needed for an accurate diagnosis. However, the concentration of microbes in the blood is extremely low in these patients (1–100 CFU/mL), traditionally requiring growth (blood culture) or amplification (*e.g*., PCR) for detection. Current culture-based methods can take a minimum of two days, while faster methods like PCR require a sample free of inhibitors (*i.e.*, blood components). Though commercial kits exist for the removal of blood from these samples, they typically capture only DNA, thereby necessitating the use of blood culture for antimicrobial testing. Here, we report a novel, scaled-up sample preparation protocol carried out in a new microbial concentration device. The process can efficiently lyse 10 mL of bacteremic blood while maintaining the microorganisms’ viability, giving a 30‑μL final output volume. A suite of six microorganisms (*Staphylococcus aureus*, *Streptococcus pneumoniae*, *Escherichia coli*, *Haemophilus influenzae*, *Pseudomonas aeruginosa*, and *Candida albicans*) at a range of clinically relevant concentrations was tested. All of the microorganisms had recoveries greater than 55% at the highest tested concentration of 100 CFU/mL, with three of them having over 70% recovery. At the lowest tested concentration of 3 CFU/mL, two microorganisms had recoveries of *ca*. 40–50% while the other four gave recoveries greater than 70%. Using a Taqman assay for methicillin-sensitive *S*. *aureus* (MSSA)to prove the feasibility of downstream analysis, we show that our microbial pellets are clean enough for PCR amplification. PCR testing of 56 spiked-positive and negative samples gave a specificity of 0.97 and a sensitivity of 0.96, showing that our sample preparation protocol holds great promise for the rapid diagnosis of bacteremia directly from a primary sample.

## Introduction

Bacteremia is defined by the presence of circulating bacteria in the blood and is detected when those bacteria are cultivatable in a blood culture. Appropriate patient care requires the assessment of this condition as quickly and precisely as possible [1]. Upon showing symptoms, most patients begin an empiric antibiotic regimen that kills a wide range of microorganisms because the specific pathogen is rarely known so early in the diagnosis. The antibiotic therapy is later scaled back or fine-tuned to a narrow spectrum once the microorganisms present in the bloodstream have been isolated and identified, typically after 2–3 days. However, it has been found that each hour that passes prior to effective antimicrobial therapy increases mortality by 5–10% [2]. Thus, reducing the delay time between symptom onset and appropriate antibiotic administration is imperative for improved patient care [2,3].

Traditionally, blood cultures are used to identify the pathogen present and are considered the gold standard for the diagnosis of bacteremic patients. Blood cultures provide unambiguous etiology of the infection and (following subculture) purified colonies for antimicrobial susceptibility testing. However, obtaining these colonies takes 2–3 days. Although this approach is effective, it is too slow and can lead to delayed and inappropriate treatment that can result in increased antibiotic resistance, longer lengths of stay in the hospital, and increased morbidity and mortality [4,5,6,7,8,9,10].

Since several days are required for the recovery and identification of the microorganism from blood culture, other rapid identification methods that do not require culturing have emerged [11,12,13,14]. Molecular methods, including microarrays and the polymerase chain reaction (PCR), can provide results in 6–8 hours [15,16,17,18,19]. Although PCR frequently has a higher rate of positivity than blood culture, PCR can remain negative, even in severe cases [20,21]. And because the corresponding sample preparation techniques do not provide viable microbes for antimicrobial testing, molecular tests must still be run side-by-side with slower blood culture methods. Researchers have recently turned to nucleic acid tests (microarrays) for host factors to detect the onset of sepsis [15,17,22]. Though these methods may be faster, they fail to give information about the specific pathogen and/or the appropriate treatment, necessitating that they be used in conjunction with other tests.

While a few molecular techniques can utilize very small volumes of whole blood for analysis [23,24,25,26,27,28], most assays need the blood components removed to analyze the microorganism’s DNA. This is because the blood components can inhibit or interfere with the analyte’s detection [20,21,29,30]. Although commercially available sample preparation kits exist for almost every type of cell-based solution, there is still a need for this procedure when diagnosing bacteremia. The ideal sample preparation method would circumvent blood culture and give purified, concentrated, viable microorganisms to allow for a wide range of downstream analysis techniques, including antimicrobial testing.

One approach to isolate viable microorganisms is the preferential lysis of blood components over microbes, though this can be quite challenging. A single milliliter of whole blood contains five billion red blood cells (45% of blood by volume) that harbor hemoglobin and seven million white blood cells that contain other proteins and DNA, all of which can interfere with PCR. Moreover, an additional 250 million platelets and innumerable free-floating plasma proteins reside therein and can also hinder downstream analyses. In the midst of all of these blood components, there are only *ca*. 100 microbes per milliliter of infected blood.

Efforts to implement preferential lysis followed by filtration of whole blood samples were published in the 1970s [31,32]. Many of these lysing solutions were deleterious to most bacteria, particularly Gram-negative bacilli, and it was found that most of these methods were too toxic to the bacteria for routine clinical use. Others concentrated viable microorganisms from whole blood using centrifugal gradients (rather than lysis) and filtration to separate the microbes from the blood components, giving recoveries that ranged from 34–107% [33,34,35]. While these methods do not require mammalian cell lysis, they do require an overnight (18 h) incubation of the filters to obtain purified colonies prior to analysis.

Loonen *et al*. recently published a sample preparation protocol that focuses on the enrichment of intact microorganisms from 1–5 mL of whole blood [36]. Their method utilized a detergent to selectively lyse the blood components and high pH to destroy the released eukaryotic DNA. The sample was neutralized and the resulting pellet was washed. The bacteria was then lysed and loaded onto a spin column for DNA extraction and purification. The researchers reported that five out of six samples spiked with 1 CFU/mL of *S*. *aureu*s gave positive results by RT-PCR.

Here, we detail a novel sample preparation protocol that differentially lyses the blood components from a relatively large sample of whole blood (10 mL), while simultaneously preserving the viability of microorganisms and concentrating them into a small volume using a custom-made concentrator device. The final result is a small, 30-μL sample, largely free of blood debris that contains almost all of the microorganisms present in the original 10-mL volume. This pellet can be streaked onto a plate to give isolated colonies that do not require further purification (avoiding both blood culture and subculture), and can also be used directly for downstream analyses. We show that the processed sample is clean enough to be added directly to nucleic acid tests, and believe that our process could be used in conjunction with other identification procedures that require clean, concentrated, viable microorganisms.

## Materials and Methods

### Devices

**Fig 1:**
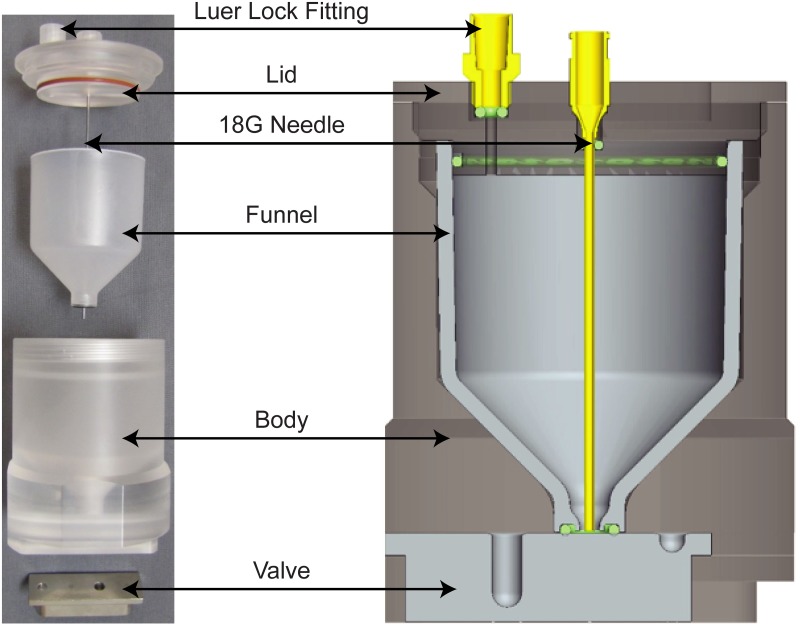
Images of the custom-made microbial concentration devices. Left: a blown-up view of the individual parts of the device. Right: a CAD model of a fully assembled device.

Custom-made microbial concentration devices were designed, fabricated, and tested (Fig. 1). The device’s body is made of the translucent material, acrylic (MSC Industrial Supply Company), and accommodates a screw-on lid, a stainless steel T-shaped slider valve, and a molded polypropylene funnel (Protomold). The 53-mL funnel serves as the device’s main chamber and feeds into the collection wells in the slider valve. The slider valve contains two smaller compartments: a 200-μL well for collecting the sample during the lysis process and a 30-μL well for collecting the final sample at the end of the lysis procedure. The lid has two holes: one for an 18 G needle used to introduce solutions and mix between steps, and another for ventilation. Silicone and *ethylene propylene diene M*-class rubber (EPDM) o-rings are used to seal interfaces. They can be seen as the red ring (silicone) around the lid and the black ring (EPDM) at the base of the funnel in the photograph, and as green cross-sectioned circles in the CAD model (Fig. 1).

### Microorganisms

We tested the recoveries of six microorganisms using our sample preparation protocol. All stock cultures were prepared by inoculating 25 mL of appropriate culture broth with a single colony (see Table 1). After inoculation, stock cultures were incubated overnight with shaking at 160 RPM and used in experiments approximately 18–24 h later. Prior to each day’s experiments, dilution series were plated onto agar to determine the number of viable organisms added to each device.

**Table 1:** List of microorganisms used in the current study and their cultivation conditions.

Microorganism	ATCC #	Culture Broth	Agar Plates	T (°C)	CO_2_
*Staphylococcus aureus* ^*a*^	29213	Tryptic Soy	Luria Bertani	37	0%
*Streptococcus pneumoniae* ^*a*^	49619	Todd Hewitt	Trypticase Soy + 5% Sheep Blood	35	5%
*Escherichia coli* ^*b*^	25922	Brain Heart Infusion	Trypticase Soy + 5% Sheep Blood	37	0%
*Haemophilus influenzae* ^*b*^	49247	Enriched Brain Heart Infusion	Chocolate II	35	5%
*Pseudomonas aeruginosa* ^*b*^	27853	Brain Heart Infusion	Trypticase Soy + 5% Sheep Blood	37	0%
*Candida albicans* ^*c*^	18804	Yeast Extract Peptone Dextrose	Sabouraud Dextrose	37	0%

^*a*^ Gram-positive bacterium;

^*b*^ Gram-negative bacterium;

^*c*^ Yeast

### Blood

Whole human blood was obtained tained from Biological Specialty Corporation (Colmar, PA) each week. Three draws of 150 mL were collected from healthy donors with the same blood type. The samples were pooled and treated with K3-EDTA as an anticoagulant.

### Procedure

**Fig 2:**
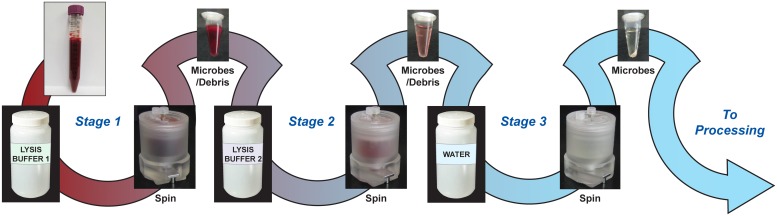
Schematic showing the sample preparation protocol with pellets removed from the valve wells at each step for visualization. Whole blood is taken through two stages of selective lysis and one wash step, leaving microorganisms intact, viable and ready for downstream analysis.

The basic outline of the sample preparation protocol is illustrated in Fig. 2. It is a three-stage process where the pellet, containing concentrated microorganisms and any blood debris, is protected in the unique T-shaped slider valve during the removal of the supernatant. The valve is then actuated, revealing the pellet to the subsequent lysis solution into which the pellet is resuspended. Each stage contains a different solution to aid in the lysis of the blood or the blood debris in the sample (see below for details). At the end of Stage 3, the pellet consists of the viable microorganisms along with any residual blood debris that may be present.

### Preparation

Prior to experiments, all device components were sterilized with 10% bleach and rinsed with sterile water. The molded funnels were then submerged in a beaker of 0.05% (w/v) Pluronic F-127 (Sigma Aldrich) to minimize microbial adherence during the procedure [37]. This beaker was placed in a sonic bath for 10 min to help facilitate adsorption of the Pluronic onto the parts’ surfaces. The Pluronic-coated components were placed in a biosafety cabinet under UV light for an hour prior to sterile assembly.

### Stage 1

Ten milliliters of pooled whole blood, spiked with *ca*. 3–100 colony forming units (CFUs) per milliliter, were added to each device through a needle inserted into the screw-on lid. Next, 2 mL of 1% (w/v) sodium polyanethol sulfonate (SPS, Acros Organics) were added. SPS is an agent that has been shown to inhibit pathways of the complement immune system which could attack microorganisms present in the blood [38,39]. DNase (4800 units) was added to the device to eliminate any free DNA that may be present in the sample along with a 1.5-mL aliquot of reaction buffer (90 mM Tris HCl pH 7.0, 27 mM MgCl_2_, 4.5 mM CaCl_2_) that provided the correct ionic environment for optimal DNase activity. Once the DNase and reaction buffer were added, the device was incubated for 10 min at 37°C on an orbital shaker at 100 RPM.

Following incubation, 40 mL of a 50 mM NH_4_Cl and 10 mM KHCO_3_ solution were added to lyse the blood components [30,40]. Next, the device was gently shaken at 100 RPM for 5 min, and then centrifuged for 20 min at 4000 RPM (3200 x *g*). This centrifugation caused the microorganisms, along with heavy components of the lysed blood, into the 200-μL well in the valve. The valve was then actuated to protect the sample while the lysis buffer was aspirated out of the device. After the solution was removed, the valve was actuated again to expose the pelleted sample to the chamber.

### Stage 2

Once the pellet was exposed, the empty funnel was filled with 40 mL of 1.75 mM CHAPS (3-[(3-cholamidopropyl)dimethylammonio]-1-propanesulfonate, Sigma-Aldrich), 2 mL of 1% SPS, 1.5 mL of reaction buffer (as described in Stage 1) and 19,200 units of DNase. CHAPS is a zwitterionic detergent that lyses blood cells without reducing bacterial viability [41]. A 60-mL syringe attached to the 18 G needle was then used to resuspend the pelleted sample in the lysis solution. Following gentle shaking at 37°C for 10 min, the device was again centrifuged for 20 min at 4000 RPM (3200 x g). Once the centrifugation was complete, the valve was actuated to protect the sample while the 2^nd^ Stage lysis buffer was removed. The valve was then actuated to expose the pelleted sample.

### Stage 3

The empty chamber was filled with 40 mL of sterile deionized water and the pellet was resuspended in the water. Before centrifugation, the valve was actuated to align the 30-μL well with the chamber. The device was spun again for 20 min at 4000 RPM (3200 x g). When the centrifugation was complete, the device was placed in the biosafety cabinet to perform the last actuation of the valve to reveal the final sample. The entire 30-μL sample, as well as 70 μL of water used to rinse the well, were transferred to a 0.5-mL tube and spun briefly in a fixed-angle rotor centrifuge prior to analysis.

### Recovery calculations

Upon completion of the experiment, the contents of the 0.5-mL tube were streaked onto agar plates. These were then incubated at the appropriate conditions overnight to allow for the formation of visible colonies (see Table 1), which were counted the next day. Prior to each experiment, the inoculation volume of diluted microbes was plated to determine the number of microorganisms added to the devices. When the inoculation quantity was compared to the recovered quantities, it was possible to calculate the percentage of viable pathogens recovered from each experiment. It is worth noting that microorganisms can multiply over the course of an experiment, and all recovery values contain a certain amount of error. As a result, it is possible to obtain recoveries greater than 100%.

### RT-PCR

For positive samples, 10 mL of whole blood were spiked with methicillin-sensitive *S*. *aureus* (MSSA) to give a bacterial concentration of *ca*. 100 CFU/mL. Equivalent volumes of unspiked whole blood served as negative controls. Multiple positive and negative samples were run side-by-side through the sample preparation protocol. From each device, 100 μL were pipetted gently to resuspend the pellet (30 μL from valve plus the 70-μL well wash) and 60 μL were plated for quantitation. To the remaining 40-μL sample, 2 μL of lysostaphin (100 μg/mL in water) were added. The sample was vortexed and then incubated at 37°C for 30 min. Following lysostaphin treatment, the samples were boiled at 95°C for 5 min and spun down briefly.

Gene Expression Master Mix from Life Technologies and a Taqman assay designed by ABI Primer Express to target the *S*. *aureus tufA* gene were used for real-time PCR experiments (Forward: 5′-CATGGTTGACGATGAAGAATTATTAGA; Reverse: 5′-TGGGAAGTCATATTCGCTTAATAAGTC; Probe: 5′-FAM-AGTAGAAATGGAAGTTCG-MGB). Four microliters of resuspended lysostaphin-treated samples (*ca*. 6% of the total sample) were added to PCR wells in triplicate to obtain a total reaction volume of 30 μL. Following a 10-min 95°C activation step on an Applied Biosystems 7500 Real-Time PCR System, the samples were cycled forty times between 95°C and 60°C for 15 seconds and one minute, respectively. Calibration curves of gDNA were included for each experiment to determine the efficiency of the PCR. A sample was designated as positive if two or three of the triplicate wells gave amplification and as negative if zero or one well amplified over the 40 cycles.

## Results and Discussion

### Process development

In the development of our reported protocol, several reagents were vital to achieving our goal of selectively lysing 10 mL of whole blood while collecting viable microorganisms. Pluronic F-127 aided our sample preparation protocol by preventing microorganisms from adhering to the funnels during processing. Pluronic F-127 is a linear triblock co-polymer of poly(ethylene oxide) (PEO) and poly(propylene oxide) (PPO). When it adsorbs onto polypropylene, the hydrophobic PPO center adsorbs to the surface while the hydrophilic PEO chains are suspended in solution, creating a brush conformation [42]. The brush conformation of PEO yields non-adhesive properties due to its highly hydrated polymer chains. Previous experiments have shown that Pluronic F-127 treatment reduces bacterial adhesion to plastic surfaces [37].

Lysis buffers were selected from lysis procedures found in the literature and then optimized to our sample volume (10 mL whole blood) since most published lysis procedures have been developed for much smaller blood volumes. In the 1^st^ Stage of the protocol, we use a blood cell lysis buffer that predominately lyses red blood cells since they are the principal component of whole blood; white blood cells are lysed as well. Once the majority of the blood cells (both red and white) are eliminated, any extraneous proteins and cell membranes (erythrocyte ghosts) are solubilized using our 2^nd^ Stage lysis buffer. Water is used in the 3^rd^ Stage to collect the bacteria into a solution that is friendly to many downstream analysis techniques. PBS could also be used if the subsequent analysis technology can tolerate it.

DNase was utilized to eliminate free DNA produced during the processing. In blood, DNA is typically found within white blood cell nuclei as tightly compacted chromosomes. When white blood cells lyse, their chromosomes unravel and the DNA expands greatly to form a gel-like substance potentially capable of trapping the microorganisms. DNase was used to break down this gel-like substance and prevent such trapping. Ten milliliters of blood can contain between 300–600 μg of DNA. To digest that much material, it was necessary to use at least 300 units of DNase. However, when that quantity was used, gel (that stained positive for DNA) still remained. The DNase concentration was therefore incrementally increased until no DNA was present (as determined by gel electrophoresis) and the gel was no longer observed (4800 units and 19,200 units for Stages 1 and 2, respectively). The high concentration of DNase is likely to be necessary in a clinical setting, where the blood specimens are expected to have elevated white blood cell counts and therefore more DNA in the samples.

### Bacterial recoveries

**Fig 3:**
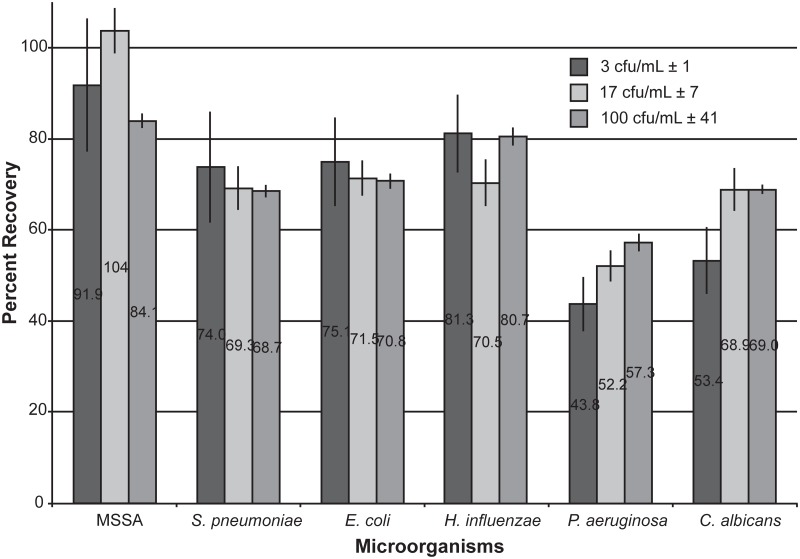
Graph showing the percent recoveries of viable microorganisms following our sample preparation protocol. Data are averages of several replicates (n>3).

Using our sample preparation protocol, we were able to successfully recover viable microorganisms from 10 mL of whole blood within a range of clinically relevant concentrations (Fig. 3). At a concentration of 100 CFU/mL, all microorganisms had a recovery greater than 55%, with methicillin-sensitive *Staphylococcus aureus* (MSSA), *Escherichia coli* and *Haemophilus influenzae* all greater than 70% recovery. At the lowest concentration tested of 3 CFU/mL, MSSA, *E*. *coli*, *Streptococcus pneumoniae*, and *H*. *influenzae* all had greater than 70% recoveries that, according to unpaired t-tests, were not statistically different from their recoveries at higher microbial concentrations (*p*>>0.05). *Pseudomonas aeruginosa* and *Candida albicans* had 43–53% recoveries, which were significantly different from their recoveries at 100 CFU/mL (*P*. *aeruginosa*: *p* = 0.005; *C*. *albicans*: *p* = 0.002).

It was hypothesized that the thick cell wall of Gram-positive bacteria would make these organisms less prone to lysis during the processing compared to Gram-negative bacteria. However, the high recoveries of Gram-negatives *E*. *coli* and *H*. *influenzae* show that there are other factors at play. When the shape of the microorganisms was considered, those that are spheroid (MSSA, *S*. *pneumoniae*, and *H*. *influenzae*) seem to have a slight advantage over those that are rod-shaped (*E*. *coli*, *P*. *aeruginosa*). It could be postulated that rod-shaped bacteria rupture in their elongated midsections more frequently than at their dense rounded ends, making them more susceptible to lysing than spheroid shaped bacteria.

The bacterial recoveries were analyzed using a Bayesian analysis algorithm to determine the probability of an event. In this case, we wanted to determine the probability that a recovery of greater than or equal to 40% would occur when given a 10-mL blood sample consisting of bacteria at a random (but known) concentration. Using Bayesian analysis, it was ascertained that all of the microorganisms we tested at each selected concentration had 100% probability that their recovery would be greater than 40%.

We note that only viable microorganisms are represented in our recoveries, as these values are calculated via quantitative plating. Because of this, intact but non-viable microorganisms are unaccounted for. If downstream nucleic acid tests were used, these microbes would contribute to a positive result even though they would produce no colonies on plates. However, we are unable to quantify these microbes because purifying the original sample prior to PCR introduces additional error due to incomplete DNA recovery.

### Pellet analysis

**Fig 4:**
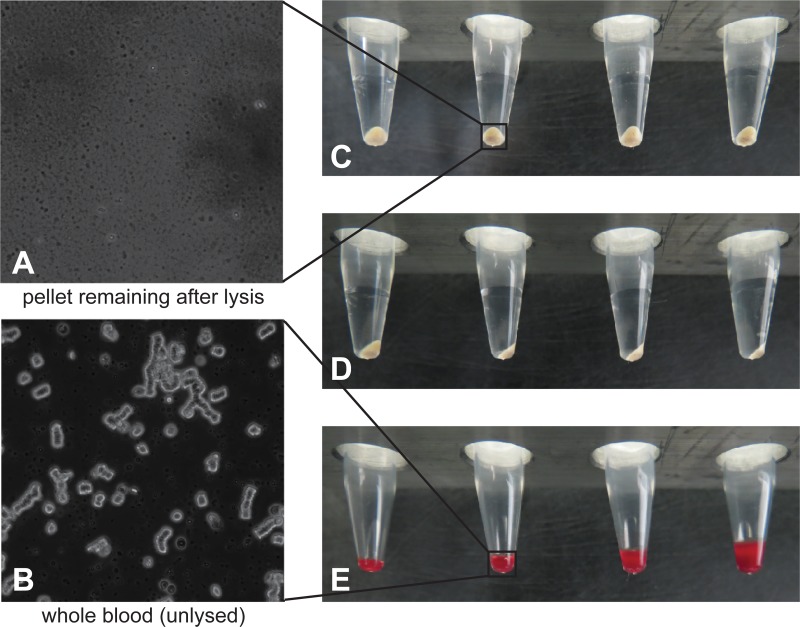
Images of the pellets after lysing 10 mL of whole blood. A: Microscopic view of an unspiked blood sample after lysis process at 60x magnification; B: For reference, microscopic view of unlysed whole blood at 60x magnification; C: Front view of the pellets; D: Side view of the pellets; E: For reference, from left to right, 2.5, 5.0, 10, and 15 μL of whole blood.

At the end of the process of lysing 10 mL of whole blood, a small pellet with a volume of approximately 5 μL remains, as shown in Fig. 4. Several biochemical methods were used to analyze the pellet’s contents. The pellets were examined spectrally, using a spectrophotometer and a nephelometer. Although no discernible features were identified in the traditional UV-vis mode, there were weak, but identifiable bands using the nephelometer. A very small peak corresponding to the Soret band of the heme group indicated that the heme co-factors in the hemoglobin were almost completely destroyed. Several minute bands correlated to absorption of proteins and cell membrane lipids (data not shown). A Western blot labeled with anti-human serum albumin was performed to further characterize the residual proteins. The blot showed that most of the human serum albumin was removed during processing, but some unidentified protein fragments remained (data not shown).

Additionally, microscopic images were taken of pellet smears stained with Diff-Quik to determine the contents of the pellet. Although no distinguishable structures were observed microscopically, some structures vaguely resembled a polymer. These shapes could indicate that there was an incomplete degradation of free DNA; however, gel electrophoresis and nucleic acid staining of the pellet confirmed that the DNA had been fully digested by the enzymatic treatments of Stages 1 and 2 (data not shown).

After analysis with a variety of techniques, the composition of the pellet remained only partially identified. Since the pellet must be composed of cell debris, we posit that the residual debris consists primarily of aggregated proteins and lipid membranes. Nonetheless, the process is able to efficiently remove the vast majority of both solid and liquid components of the 10 mL of whole blood, resulting in a 5-μL pellet consisting of viable microorganisms (if present in the original sample) and a small volume of remaining cell debris. Because the cell debris has the potential to interfere with downstream detection schemes, one inhibitor-sensitive detection technique, RT-PCR, was tested.

### RT-PCR

With our protocol optimized, we set out to couple it with a rapid detection method. We employed a Taqman assay that amplifies *tufA* in *S*. *aureus* for this purpose. Initial tests showed minimal PCR amplification of the processed pellets, indicating that the recovered bacteria were still intact and their DNA was inaccessible (data not shown). To make the bacterial DNA available for amplification, we chose to employ an enzymatic lysis step that would break down the thick cell walls of Gram-positive bacterium. Aliquots (4 μL) of these lysostaphin-treated pellets were pipetted directly into triplicate wells for PCR analysis.

**Fig 5:**
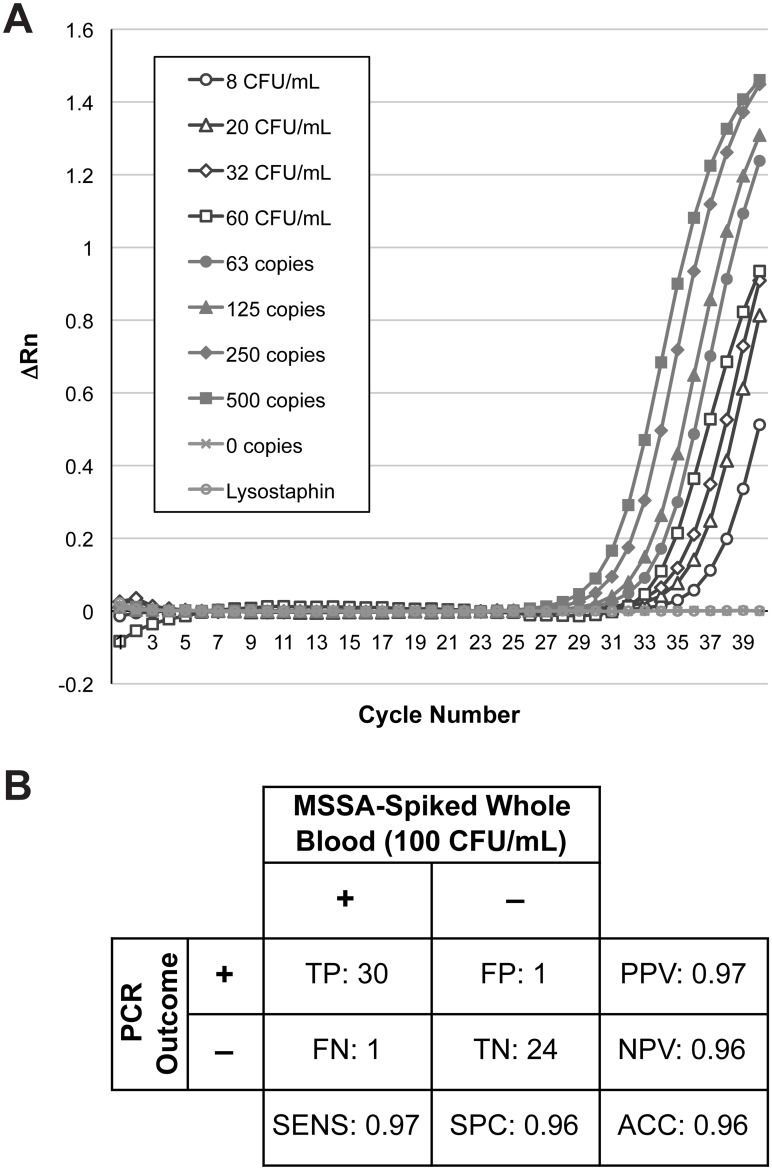
Results from PCR. A: PCR data show that pellets from whole blood samples (10 mL) spiked with higher concentrations of MSSA amplify sooner (pellet volume in PCR well = 8 μL). B: Contingency table for PCR analysis of processed positive (100 CFU/mL) and negative (0 CFU/mL) whole blood samples.

In a blinded study, 31 spiked and 25 unspiked whole blood samples from healthy donors were run side-by-side through our sample preparation protocol and then analyzed by PCR (Fig. 5). The positive samples (n = 31) were spiked with a final *S*. *aureus* concentration of *ca*. 100 CFU/mL while the negative samples (n = 25) contained no bacteria. The observed Ct values for the positive samples were 34.0 ±2.0. The sensitivity of the entire process was 0.97 and the specificity was 0.96, with only one false positive and one false negative occurring overall (Fig. 5). These data suggest that lysostaphin treatment is suitable for *S*. *aureus* nucleic acid detection and that the pellet contents do not inhibit the sensitive PCR assay.

### Conclusions

We have detailed a novel sample preparation protocol that utilizes a custom-made concentration device. We have shown that our procedure differentially lyses blood components from a relatively large sample of whole blood (10 mL), while simultaneously preserving the viability of microorganisms and concentrating them into a small volume. The final result is a 30-μL sample (*ca*. 5 μL solid material), largely free of blood components, which contains almost all of the microorganisms present in the original 10-mL volume. Others have recently published efficient protocols for enriching intact microorganisms from 5 mL of whole blood [36]. However, the viabilities of the enriched microbes are not discussed, and pathogen DNA from the resulting pellets (of unknown size) must be purified on a spin column prior to PCR. Here, we have shown that our processed sample can be split for both immediate analysis and to obtain isolated, purified colonies (following overnight incubation). It is clean enough to be used for a variety of downstream assays, including those that require purified, concentrated, viable microorganisms.

Novel optical spectroscopy identification techniques, such as surface enhanced Raman spectroscopy (SERS), that produce species-specific spectra for the detection of clinically-relevant microorganisms [43,44,45,46,47] may replace culture-based testing in the future. When coupled with rapid detection technologies, we envision that the reported sample preparation protocol could be utilized as a complete rapid diagnostic for bacteremic patients; starting with just 10 mL of whole blood and ending with a definitive identification of the pathogen in less than two hours. Work is currently being completed to simplify the manufacturing of the concentrator devices and to bring this complete diagnostic to fruition.
